# Properties of *Salvia officinalis* L. and *Thymus serpyllum* L. Extracts Free and Embedded into Mesopores of Silica and Titania Nanomaterials

**DOI:** 10.3390/nano10050820

**Published:** 2020-04-25

**Authors:** Ana-Maria Brezoiu, Mioara Prundeanu, Daniela Berger, Mihaela Deaconu, Cristian Matei, Ovidiu Oprea, Eugeniu Vasile, Ticuța Negreanu-Pîrjol, Delia Muntean, Corina Danciu

**Affiliations:** 1 Department of Inorganic Chemistry, Physical-Chemistry & Electrochemistry, Faculty of Applied Chemistry and Materials Science, University “Politehnica” of Bucharest, 1-7 Gheorghe Polizu Street, 011061 Bucharest, Romania; anamaria_brezoiu@yahoo.com (A.-M.B.); mioara_prundeanu@yahoo.com (M.P.); daniela.berger@upb.ro (D.B.); mihaela_deaconu@yahoo.com (M.D.); cristian.matei@upb.ro (C.M.); ovidiu.oprea@upb.ro (O.O.); 2 Department of Oxide Materials Science and Engineering, Faculty of Applied Chemistry and Materials Science, University “Politehnica” of Bucharest, 1-7 Gheorghe Polizu Street, 011061 Bucharest, Romania; 3 Faculty of Pharmacy, “Ovidius” University of Constanta, Aleea Universitatii No. 1, 900470 Constanta, Romania; ticuta_np@yahoo.com; 4 Department of Microbiology, University of Medicine and Pharmacy “Victor Babes”, Eftimie Murgu Square No. 2, 300041 Timisoara, Romania; muntean.delia@umft.ro; 5 Department of Pharmacognosy, University of Medicine and Pharmacy “Victor Babes”, Eftimie Murgu Square No. 2, 300041 Timisoara, Romania; corina.danciu@umft.ro

**Keywords:** mesoporous silica, titania, polyphenolic extract, *Salvia officinalis*, *Thymus serpyllum*, encapsulated extract

## Abstract

This study evidenced the nanoconfinement effect on polyphenolic extracts prepared from *Salvia officinalis* L. and *Thymus serpyllum* L. into the mesopores of silica and titania nanomaterials on their radical scavenging capacity and antimicrobial potential. The ethanolic and hydroalcoholic extracts obtained either by conventional or microwave-assisted extraction were characterized in terms of total polyphenols, total flavonoids, and chlorophyll content, as well as radical scavenging activity by consecrated spectrometric determinations. The phytochemical fingerprint of extracts was analyzed by high-performance liquid chromatography-photodiode array detector. *Salvia officinalis* extracts exhibited better radical scavenging capacity and antimicrobial potential than *Thymus serpyllum* extracts. The mesoporous MCM-41 silica and titania nanomaterials, prepared by the sol–gel method, were characterized by small- and wide-angle powder diffraction, FTIR spectroscopy, nitrogen adsorption–desorption isotherms, scanning electron microscopy and transmission electron microscopy, while the materials containing embedded extracts were analyzed through Fourier-transform infrared spectroscopy, N_2_ sorption measurements, and thermal analysis. All extracts free and embedded in mesoporous matrix exhibited high radical scavenger properties and good bactericidal activity against several reference strains. It was proved that by embedding the polyphenolic extracts into mesopores of silica or titania nanoparticles, the phytochemicals stability was enhanced as the materials containing extract exhibited higher radical scavenger activity after 3–6 months storage than that of the free extracts. Additionally, the extract-loaded material showed mild improved antimicrobial activity in comparison with the corresponding free extract.

## 1. Introduction

The oxidative stress caused by the imbalance between reactive oxygen species and antioxidants contributes significantly to the alteration of human health condition. Antioxidants are compounds able to quench reactive oxygen species and are valuable in preventing some diseases. Polyphenols are the main class of phytochemicals found in plants, which are natural antioxidants and some of them have significant antimicrobial activity [1,2,3,4,5]. Due to intensive and inappropriate use of antibiotics, many pathogens become drug resistant and a real threat for human health. Thus, there is a growing interest in finding new compounds with bactericidal properties, one of the options being the recovery of some natural compounds from plants. Polyphenolic extracts prepared from various medicinal herbs exhibit antioxidant, antimicrobial, anti-inflammatory, or even antitumoral properties and their benefits for human health are well known [6,7].

*Salvia officinalis* L. (common sage; *Lamiaceae* family) is listed among the plants qualified as rich in bioactive compounds, polyphenols and flavonoids being responsible for its high radical scavenger activity [8,9]. It is widely used for both culinary and medicinal purposes, in the production of a wide range of phytopreparations, like tea mixtures, supplements with therapeutic benefits in serious diseases such diabetes [10], Alzheimer’s [11], or cancer [12], and food preservation additives [13]. Additionally, *Salvia officinalis* extracts exhibit good antimicrobial and antifungal activities on various strains and fungi, respectively, which were reported to be associated with the presence of carnosol or carnosic acid in the extracts [9]. Based on antimicrobial activity of some natural compounds from plants, the use of polyphenolic extracts as natural food preservatives is of growing interest in order to extend the shelf life in a natural manner of foods through reducing the microbial load, thus improving their quality and safety for consumption. Recently, some preparations based on common sage were tested to prolong the frozen storage of chicken meat at low pressure [13].

*Thymus serpyllum* (wild thyme; *Lamiaceae* family) is an aromatic flowering plant having high levels of essential oils and polyphenolic compounds, which are either phenolic acids (e.g., chlorogenic, caffeic, and rosmarinic acids) or flavonoids (e.g., luteolin and apigenin glucuronide) reported as responsible for high radical scavenger potential and anti-inflammatory activity [14]. Recently, wild thyme extracts were assessed for the treatment of human breast cancer and among tested polyphenols, rosmarinic acid proved to be the most efficient, the viability of MCF-7 cells resistant to adriamycin being reduced with 14% when a concentration of 1.25 mM was applied [15].

It is well known that the conditions of the extraction process like contact time, solvent/plant ratio, solvent nature, temperature, irradiation with microwaves, or ultrasounds, greatly influence the recovery of valuable bioactive compounds [3,16,17,18]. For instance, a recent report showed an increase by 2.1 times of total phenolic content and 2.2 times of radical scavenger activity of Dalmatian sage extracts through the extraction assisted by high voltage electrical discharge [16]. Nicolai et al. reported the preparation of *Salvia officinalis* extract by ultrasounds extraction in ethanol at 35 kHz with an input power of 320 W, which contained 1.25 ± 0.09 mM rosmarinic acid corresponding to an extract concentration of 0.1 mg/mL and the extract exhibited a very good radical scavenging properties [17].

Many factors, like pH, oxygen availability, temperature, light exposure, and metal ions can affect the polyphenols stability. The phytochemicals from polyphenolic extracts can oxidize when hydroxyl groups in ketone form cannot donate hydrogen atoms, so the polyphenols stability decreases with the number of hydroxyl groups increases [19]. The stability of phenolic compounds can be affected by epimerization, auto-oxidation, or esterification/alkylation reactions of polyphenols hydroxyl groups [20]. 

Herein, we emphasize the role of mesoporous inorganic materials, silica, and titania as support for polyphenolic extracts on improving the natural compounds stability. We compare the properties of polyphenolic extracts prepared in different conditions from two plants from the *Lamiaceae* family, *Salvia officinalis,* and *Thymus serpyllum*, free and embedded into mesoporous inorganic matrices. To the best of our knowledge, it is the first report with respect to the nanoconfinement effect of extracts into mesopores of silica and titania nanomaterials on their radical scavenging and antimicrobial properties. Khan et al. studied the adsorption of quercetin and rutin into the pores of titania surface functionalized mesoporous silica and showed that radical scavenger activity of bounded or recovered quercetin from mesoporous matrix, determined using 2,2–diphenyl–1–picrylhydrazyl radical (DPPH) assay is at least 80% of the pure compound [21]. We choose these two inorganic mesoporous matrices because both silica and titania exhibit low toxicity and good biocompatibility being accepted as food and pharmaceutical additives. Moreover, they are considered safe and inert by FDA (US Food and Drug Administration) [22,23]. Mesoporous silica has high porosity that can be exploited to confine in its pores a high amount of organic compounds [24], the interactions between phytochemicals and pristine silica surface being weak, especially hydrogen or van der Waals intermolecular forces. Unlike silica, mesoporous titania has lower porosity, but can interact more strongly with bioactive compounds through donor–acceptor bonds. Another purpose in choosing titania was related to the efficiency of titania coatings in destroying of bacterial and fungus biofilms of pathogens like *Streptococcus*, *Listeria monocytogenes*, *Candida albicans* [25,26,27], and *Pseudomonas aeruginosa* [28].

## 2. Materials and Methods 

### 2.1. Materials

For mesoporous inorganic matrices, tetraethyl orthosilicate (TEOS, Fluka, Seelzer, Germany ), trimethylhexadecylammonium bromide (CTAB, Alfa Aesar, Ward Hill, MA, USA), 25% ammonia aqueous solution (Scharlau, Scharlab S.L., Barcelona, Spain), titanium(IV) isopropoxide (>97%, Aldrich Chemical Co Inc., Milwaukee, WI, USA), poly(ethylene glycol)–block–poly(propylene glycol)–block–poly(ethylene glycol), MW = 5800 (Pluronic, P123, Aldrich Chemical Co Inc., Milwaukee, WI, USA), acetic acid (>99.7%, Sigma-Aldrich Co. Merck Group, Darmstadt, Germany), and 2–propanol anhydrous (99.5%, Sigma-Aldrich Co., Merck Group, Darmstadt, Germany) were used as received.

Folin–Ciocalteu reagent, 2,2–diphenyl–1–picrylhydrazyl (DPPH), 6–hydroxy–2,5,7,8–tetramethylchroman–2–carboxylic acid (Trolox, 97%), 2,2’–azino–bis (3–ethylbenzothiazoline–6–sulphonic acid) (ABTS), sodium carbonate, potassium persulphate (K_2_S_2_O_8_), and 36.5–38% hydrochloric acid were purchased from Sigma-Aldrich Co. (Merck Group, Darmstadt, Germany). For chromatographic analyses, the following HPLC-grade compounds were used: gallic acid (Alfa Aesar, Ward Hill, MA, USA 98%), protocatechuic acid (>98%), (−)epicatechin (>98%), vanillic acid (>98%, GC-grade), ellagic acid dihydrate (>98%, HPLC-grade), chicoric acid (>98%), and trans-ferulic acid (>98%, GC) from Tokyo Chemical Industry (Tokyo, TCI, Japan), caffeic acid 98%, quercetin (>95%, HPLC-grade), rutin hydrate 95%, myricetin (>96%), rosmarinic acid (>98%), catechin hydrate, and kaempferol (>97%) from Sigma (Merck Group, Darmstadt, Germany), syringic acid (>98.5%) and caftaric acid from Molekula GmbH (Munich, Germany), trans-p-coumaric acid and trans-resveratrol (Sigma-Aldrich Co., Merck Group, Darmstadt, Germany), and chlorogenic acid from the HWI Group, Alpen Aan de Rijn, The Netherlands. The solvents used for extract or samples preparation or HPLC-PDA analyses were acetonitrile (Riedel-de Haën, Honeywell Riedel-de Haën, Seelzer, Germany), ethanol (Riedel-de Haën), formic acid (Merck Group, Darmstadt, Germany), and ultrapure water (Millipore Direct-Q3 UV water system, version Q3 UV, product no. C9185, Merck Group, Darmstadt, Germany) equipped with a Biopack UF cartridge.

Leaves of *Salvia officinalis* or *Thymus serpyllum* from the spontaneous wild flora of a hill region of Transylvania (Romania) were chosen as vegetal materials for the preparation of sage or wild thyme extracts considering their content in valuable natural compounds from polyphenols and flavonoids class.

### 2.2. Preparation of Phenolic Extracts from Salvia officinalis and Thymus serpyllum 

The ethanolic and hydroalcoholic (ethanol/water = 4/1 v/v) conventional extracts from both plants were prepared at reflux, in three extraction stages of 1 h with the separation of the vegetal material after each step, the replacement of the solvent in the same volume and then the three extracts were mixed. The ethanolic extracts from *Salvia officinalis* leaves were obtained by either conventional (Conv) or microwave-assisted (MW) extraction using a plant/ethanol ratio of 1/50 (w/v), while the hydroalcoholic extract was prepared only through conventional process at vegetal material/solvent of 1/30 (w/v). The common sage ethanolic extract was labeled So(Conv)-1, while the hydroalcoholic one was denoted So(Conv)-2. The MW ethanolic extract, So(MW)-1, was obtained using a Miniflow 200SS microwave reactor (Sairen, Décines Charpieu, France) a MW power of 75 W (reflected power of 3 W) and three extraction stages of 15 min. Both ethanolic and hydroalcoholic (ethanol/water = 4/1 v/v) extracts from *Thymus serpyllum*, labeled Ts(Conv)-1 and Ts(Conv)-2, respectively, were prepared by conventional extraction in the same conditions as for So(Conv)-2. The polyphenolic extracts were dried under vacuum until reaching a constant mass and then were redissolved for preparing extracts of a certain concentration.

### 2.3. Characterization of Polyphenolic Extracts

The ethanolic and hydroalcoholic phenolic extracts from *Salvia officinalis* L. and *Thymus serpyllum* L. were characterized by various spectrophotometric determinations (Shimadzu UV-1800, Shimadzu Corporation, Kyoto, Japan), such as total polyphenols, total flavonoids, as well as chlorophyll a (Chl a) and b (Chl b) pigments contents. The spectrometric determination of total polyphenols content using the Folin–Ciocalteu method and total flavonoids content using aluminum chloride were described elsewhere [29]. For the total polyphenols content determination, apart from the calibration curve for gallic acid, it was also used a calibration curve for caffeic acid (10–150 µg/mL domain) at both 650 nm (*y* = 0.01048 × *x* + 0.013) and 765 nm (*y* = 0.01054 × *x* + 0.017). The total flavonoids were expressed as rutin hydrate equivalents in the concentration range of 0–100 µg/mL at 410 nm maximum wavelength (*y* = 0.0135 × *x*).

For the determination of chlorophyll a and b content, two samples of each extract with different concentration were used. The UV-vis spectrum of each sample was recorded, and the solution absorbance was measured at 665 nm, 649 nm, and 750 nm. The chlorophyll a and b contents were computed using the Ritchie’s equations:

Ch-a = 13.5275 × (A_665_ − A_750_) − 5.201 × (A_649_ − A_750_) × *f* × *S* (1)

Ch-b = 22.4327 × (A_649_ − A_750_) − 7.0741 × (A_665_ − A_750_) × *f* × *S* (2)

where Ch-a—chlorophyll a in µg, Ch-b—chlorophyll b in µg, A—the solution absorbance at a certain wavelength, f—dilution factor, and S—volume of solvent (ethanol or ethanol–water = 4/1) [30].

The chemical profile of prepared extracts was assessed by reverse phase high performance liquid chromatography (HPLC; Shimadzu Nexera 2, Shimadzu Corporation) with photodiode array detector (SPD-M30A, Shimadzu Corporation), which operates in the wavelength range of 250–600 nm, using a Nucleoshell® reversed-phase C18 column (Macherey-Nagel GmbH & Co. KG, Düren, Germany) 4.6 mm × 100 mm (2.7 µm), two mobile phases: 2.5% aqueous formic acid solution (mobile phase A) and 90% aqueous acetonitrile with 2.5% formic acid (mobile phase B). The chromatograph was equipped with a LC-20ADXR quaternary pump (Shimadzu Corporation), DGU-20A5R vacuum degasser (Shimadzu Corporation), SIL-30 AC autosampler (Shimadzu Corporation) and CTO-20AC (Shimadzu Corporation) column oven. LabSolutions software Lite LC/GC software (version 5.82, Shimadzu Corporation) was used for acquiring and processing the data. The details of the elution program were described elsewhere [29]. For the polyphenols separation, identification and quantification, a gradient elution at constant flow of 0.4 mL/min, a temperature of 20 °C, and a volume of 1 µL for the injection was used.

### 2.4. Obtaining of Mesoporous Inorganic Matrices

MCM-41 mesoporous silica support, labeled MCM-41E with an ordered hexagonal pore array was obtained through the sol–gel method assisted by hydrothermal treatment through a reported procedure using tetraethyl orthosilicate as silica source, CTAB as template and concentrated ammonia aqueous solution to ensure base medium. The TEOS:CTAB:NH_3_:H_2_O molar ratio used for the silica synthesis was 1:0.147:3.19:149 [31]. The surfactant bound on silica was removed by extraction in saturated ethanolic solution of NH_4_Cl at reflux for 2 h and 1 h by ultrasounds treatment.

Mesoporous titania was also obtained by the sol–gel method based on the hydrolysis and condensation reactions that involved the cooperative assembly of titanium isopropoxide with the structure directing agent, triblock copolymer Pluronic P123, in acidic medium. Initially, 1.25 g of Pluronic P123 was dissolved in 50 mL of anhydrous 2–propanol, followed by the addition of 1.5 mL of glacial acetic acid and 3.76 mL of titanium isopropoxide. The solution was stirred at 40 °C, 1 h and then, 1 mL of water was added. The reaction mixture was aged under magnetic stirring at 40 °C, 24 h, followed by a treatment at reflux for another 24 h. The solid was filtered off, washed with ethanol and water, dried at 80 ⁰C overnight. To remove the template agent, a Soxhlet extraction in ethanol during 30 h, followed by a calcination step at 450 °C, 5 h with 0.5 °C/min heating rate were performed.

### 2.5. Embedding of Phenolic Extracts into Mesoporous Inorganic Supports

For embedding of the prepared phenolic extracts, two types of mesoporous inorganic supports, MCM-41 silica and titania, were used. The materials containing phenolic extract were obtained by incipient wetness impregnation method. Thus, the inorganic support, previously dried in vacuum at 110 °C overnight, was mixed with the polyphenolic extract having a concentration of 30 mg/mL for So(MW)-1 and So(Conv)-1, 20 mg/mL for So(Conv)-2 and 28.5 mg/mL in the case of Ts extracts, and the resulted suspension was dried in vacuum at room temperature (20 °C) for at least 8 h, under dark conditions. The materials containing embedded extract were labeled extract@mesoporous support.

### 2.6. Characterization of Supports and Materials Containing Extract

The mesoporous inorganic supports were investigated by small- and wide-angle powder X-ray diffraction, Fourier transform infrared spectroscopy (FTIR), thermal analysis, and scanning and transmission electron microscopy. The FTIR spectra were recorded on a Bruker Tensor 27 spectrophotometer (Bruker Corporation Optik GmbH, Bremen, Germany) in the wavenumber range of 4000–400 cm^−1^ using the KBr pellets technique. The small- and wide-angle X-ray diffraction (XRD) was carried out on a Rigaku MiniFlexII diffractometer (Rigaku Corporation, Tokyo, Japan) with Cu-Kα radiation. The small-angle XRD analyses were recorded in 1.2–6.0° 2θ rage using a scanning rate of 0.5°/min and a step of 0.01°, while the wide-angle powder XRD patterns were carried out in 10–70° 2θ range, with a scanning rate of 1°/min. SEM investigation of mesoporous supports was performed on Tescan (Brno, Czech Republic) Vega 3 LM electron microscope equipped with an energy dispersive X-ray (EDX) detector for the chemical composition determination, while TEM analysis was carried out on a FEI (Hillsboro, OR, USA) TECNAI F30 G^2^ S-TWIN high resolution transmission electron microscope with a field emission electron gun and a maximum accelerating voltage of 300 kV. The supports and materials containing polyphenolic extract were investigated by FTIR spectroscopy, thermal analysis (DTA-TG) and nitrogen adsorption–desorption isotherms. The FTIR spectra were performed to evidence the phenolic compounds adsorption. The thermogravimetric analyses, (STA 449 F3 Jupiter equipment from Netzsch, Selb, Germany or GA/SDTA851e from Mettler Toledo, Greifensee, Switzerland) were carried out using a scan rate of 10 °C/min, under synthetic air flow, to determine the polyphenolic compounds content into mesoporous matrices. The nitrogen adsorption–desorption isotherms (Autosorb iQ2 gas sorption analyzer from Quantachrome Instruments, Boynton Beach, FL, USA) were recorded at 77 K to determine the textural parameters of prepared materials. Prior to the isotherms recording, the supports and the samples containing extract were outgassed at 120 °C for 12 h and 35 °C for 17 h, respectively. The determined textural features were specific surface area values, *S*_BET_, calculated using the Brunauer–Emmett–Teller method in the 0.05–0.25 relative pressure range, the total pore volume, *V*_p_, measured at 0.99 relative pressure, and the average pore diameter, *d*, computed through non-local density functional theory (NLDFT) model from the adsorption branch of the corresponding isotherm.

### 2.7. Determination of Radical Scavenging Activity 

The radical scavenging capacity for phenolic extracts was determined through either DPPH or ABTS methods as Trolox equivalents and for solid samples containing embedded phenolic extract by the DPPH method. The details of applied procedures are provided elsewhere [29]. In brief, the radical scavenger activity (RSA) of the material containing extract was assessed in comparison with that of the support and free extract in the same amount as in the material containing extract using the degradation of DPPH solution as control, after 24 h. All experiments were performed in closed containers, in dark conditions. Thereafter, aliquots of free or embedded extract, support, and DPPH free radical solution were withdrawn, centrifuged for 15 minutes in the case of suspensions and then the solution absorbance was measured at 517 nm wavelength.

### 2.8. Assessment of Antimicrobial activity

The antibacterial and antifungal activity of these compounds were evaluated by the agar disk diffusion method and dilution method, according to the Clinical Laboratory and Standard Institute (CLSI) [32,33]. 

#### 2.8.1. Method of Disk Diffusion

The antimicrobial activity of prepared polyphenolic extracts was evaluated through agar disk diffusion method against nine reference bacterial and two fungus strains purchased from Thermo Fisher Scientific (Waltham, MA, USA): *Salmonella enterica* serotype typhimurium ATCC 14028, *Shigella flexneri* serotip 2b ATCC 12022, *Enterococcus faecalis* ATCC 51299, *Escherichia coli* ATCC 25922, *Pseudomonas aeruginosa* ATCC 27853, *Staphylococcus aureus* ATCC 25923, *Streptococcus pneumoniae* ATCC 49619, *Streptococcus pyogenes* ATCC 19615, *Bacteroides fragillis* ATCC 25285, *Candida albicans* ATCC 10231, and *Candida parapsilosis* ATCC 22019. Plates with Mueller-Hinton agar medium (Sanimed, Bucharest, Romania) were inoculated with 100 μL microbial suspension in sterile physiological serum equivalent of 10^8^ CFU/mL density (CFU-colony forming units). Next, 10 μL of polyphenolic extract were placed on a blank paper disk (BioMaxima, Lublin, Poland), which was deposited on the cultured medium surface. Then, the agar plates inoculated with the microbial suspension were incubated at 35–37 °C, 24 h. The antimicrobial activity was quantified by measuring the microbial growth inhibition diameter. The tests were performed in triplicate. Gentamycin or fluconazole disks (Bio-Rad, Marnes-la-Coquette, France) as positive control and a blank paper disk impregnated with the corresponding solvent as negative control were used.

#### 2.8.2. Determination of Minimum Inhibitory Concentration (MIC) and Minimum Bactericidal Concentration (MBC)

The antimicrobial properties of phenolic extracts prepared from *Salvia officinalis* and *Thymus serpyllum* were also assessed by broth dilution assay. Thus, Mueller–Hinton broth with a concentration of 200 μg/mL was prepared. A serial 2-fold dilutions in Mueller–Hinton broth (Sanimed, Bucharest, Romania) for each extract were performed. The test tubes were inoculated with 5 × 10^5^ CFU/mL and incubated at 37 °C for 24 h. The MIC values (the lowest concentration that determine no bacteria growth) were determined. Additionally, the test tubes with no visible growth were inoculated on Columbia agar +5% sheep’s blood or Sabouraud chloramphenicol agar (Sanimed, Bucharest, Romania) and then incubated at 37 °C for 24 h to determine the MBC values (the lowest concentration that killed 99.9% of the initial inoculum).

#### 2.8.3. Antimicrobial Activity of Materials Containing Extract by the Disk Diffusion Method

The antimicrobial activity of selected embedded extract was assessed by adapted disk diffusion method for solid samples against the same nine reference bacterial and two fungus strains. After activation of the microbial strains, a stock suspension of 10^8^ CFU/mL was prepared in a test tube containing 10 mL of sterile distilled water, its white level being evaluated by comparison with a standard McFarland solution of 0.5. From the stock suspension a dilution at 10^7^ CFU/mL was made. The culture medium was liquefied at 45 °C and stirred for complete homogenization. Each culture medium was seeded with 1 mL of inoculum of 10^7^ CFU/mL, stirred for homogenization so that the concentration would be 10^5^ CFU/mL. The freshly inoculated culture medium was poured into each Petri dish (15–20 mL) and left to solidify. On the surface, it was placed at equal distances, three cylinders of stainless steel, sterile, with an internal diameter of 8 mm and a height of 10 mm. In each cylinder, the weighed solid sample containing embedded extract and 200 μL dissolution medium were added. The Petri dishes were incubated at 35 °C for 24 h, after which the diameters of microbial growth inhibition were measured [34]. 

## 3. Results and Discussion

### 3.1. Characterisation of Polyphenolic Extracts by Spectrometric Determinations

The extract amount obtained from common sage or wild thyme plants, the radical scavenger activity, total polyphenols, and total flavonoids, as well as total chlorophyll content values are listed in Table 1. One can observe that the efficiency of the extraction assisted by microwaves (MW) was higher than that of conventional process demonstrated by a higher extract amount and total polyphenols content in agreement with literature data [35,36]. MW provides a supplementary energy to both extraction medium and plant that determines an efficient vegetal material heating. Furthermore, water from plants absorbs most of MW energy producing a local superheating that causes cell walls disruption, which leads to the improvement of phytochemicals recovery [37]. Additionally, the use of the ethanol–water mixture as a solvent led to an enhanced extract amount than in the case of absolute ethanol because of a higher variety of natural compounds, which can be recovered in the ethanol–water mixture apart from polyphenols.

The total polyphenols content (TPC) of extracts spectrophotometrically determined using the Folin–Ciocalteu method as both caffeic acid or gallic acid equivalents were in the range of 108.95–181.11 mg_GAE_/g extract (20.58–61.98 mg/g plant) and 154.67–155.50 mg_GAE_/g extract (18.21–22.20 mg/g plant) for *Salvia officinalis* and *Thymus serpyllum* extracts, respectively (Table 1). The values for TPC of common sage extracts were higher than that reported for: methanolic and ethanol–water 70/30 (v/v) extracts obtained by conventional and ultrasound-assisted extraction processes (61.3–79.6 mg_GAE_/g extract) [9], 70% aqueous acetonic extracts prepared at room temperature (40.5–96.2 mg_GAE_/g extract) [38], methanolic extracts (63.9–93.8 mg/g extract) [8], 80% aqueous methanolic extracts obtained by ultrasounds assisted extraction (2.80 mg/g plant) [39], and even that of the TPC value reported by Nutrizio et al. [16] (42.13 ± 1.24 mg_GAE_/g plant) for the 50% hydroalcoholic extract prepared through the process assisted by high voltage electrical discharges (but only in the case of So(Conv)-2 extract).

The common sage ethanolic extracts contained higher total polyphenols in comparison with wild thyme ethanolic extracts (Table 1) in agreement with the results obtained by Stanciu et al. [40] For the wild thyme extracts obtained by pressurized liquid extraction at different temperatures, Miron et al. [41] reported TPC values in the range of 34–78.72 mg_GAE_/g extract (for ethanolic extracts), 72.20–91.07 mg/g extract (for water extracts) and 102.20–119.95 mg/g extracts (for hydroalcoholic extracts), which are all lower than the TPC values of both ethanolic and ethanol–water extracts obtained in this work. One can notice that unlike in the case of So extracts, the TPC values for Ts ethanolic and ethanol–water extracts are very close (Table 1). The TPC value for our Ts ethanolic extract was slightly lower than the reported value by Pasca et al. for an extract prepared at room temperature for 14 days (22.670 ± 0.003 mg_GAE_/g plant) [42].

Total flavonoids content, TFC, expressed as rutin hydrate equivalents for So extracts has values between 40.92 and 75.52 mg_RE_/g extract (13.26–19.41 mg_RE_/g plant), higher amount being noticed for the ethanolic extracts, especially for that prepared by MW extraction (Table 1). In the case of Ts extracts, a higher TFC value (83.10 mg_RE_/g extract or 11.60 mg_RE_/g plant) was determined for ethanolic extract in comparison with the hydroalcoholic one (81.28 mg_RE_/g extract or 9.78 mg_RE_/g plant), but lower than that reported by Abramovic et al. for Ts ethanolic extracts prepared at 60 °C (11.00 ± 1.00 mg/g plant) [43]. The TFC values of So extracts are slightly lower than that obtained by Oniga et al. (28.2 ± 1.8 mg_RE_/g plant) [44]. Our TFC values as quercetin equivalents are in the range of 13.56–25.03 mg_QE_/g extract for So extracts, slightly lower than that reported by Duletic et al. (27.30 ± 8.48 mg_QE_/g extract) [45]. Amamra et al. reported lower TFC values for methanolic extracts of Ts (21.92 ± 1.14 mg_QE_/g extract) in comparison with our ethanolic and ethanol–water extracts (26.93–27.54 mg_QE_/g extract) [46].

**Figure 1:**
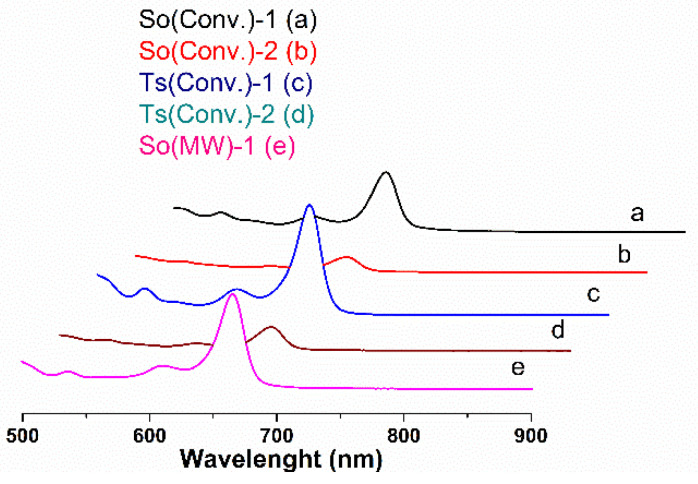
Chlorophyll spectra for: (a) So(Conv)-1, (b) So(Conv-2), (c) Ts(Conv)-1, (d) Ts(Conv)-2, and (e) So(MW)-1 extracts.

The chlorophyll content of common sage and wild thyme extracts is listed in Table 1, and the corresponding UV-vis spectra are presented in Figure 1. Chlorophyll a and chlorophyll b are pigments contained in green plants that contribute to the photosynthesis. Chlorophyll b differs from chlorophyll a only by the carbonyl group linked to the porphyrin ring and it is more soluble in polar solvents [47]. The Ts extracts were richer in chlorophyll a than the So ones. One can also noticed that the ethanolic polyphenolic extracts had higher chlorophyll a content than the ethanol–water ones. Moreover, MW treatment favored a larger content of chlorophyll in the So(MW)-1 extract than that of So(Conv)-1 sample (Table 1). Zilic et al. reported values of Ch-a and Ch-b for So extracts in 70% acetone of 213.2 ± 5.7 mg_Ch-a_/100 g plant and 102.1 ± 8.8 mg_Ch-b_/100 g plant and for Ts extracts of 147.5 ± 5.8 mg_Ch-a_/100 g plant and 68.5 ± 3.5 mg_Ch-b_/100 g plant [48], which are higher than the chlorophyll content obtained for our ethanolic extracts (58.93–101.52 mg_Ch-a_/100 g plant, 10.76–24.93 mg_Ch-b_/100 g plant for So extracts and 61.79 ± 0.00 mg_Ch-a_/100 g plant, 10.71 ± 0.35 mg_Ch-b_/100 g plant for Ts extracts. This may be explained considering that acetone is a more nonpolar solvent than ethanol and is therefore able to solubilize more chlorophyll.

### 3.2. Chemical Profile of Common Sage and Wild Thyme Extracts

**Figure 2:**
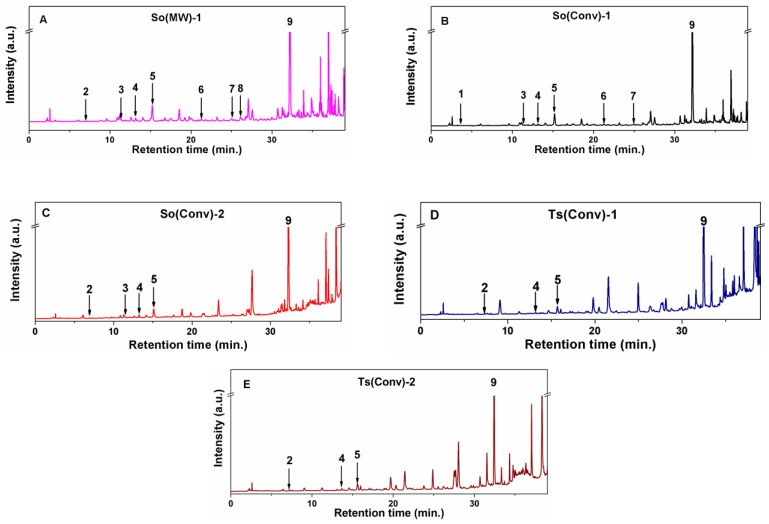
HPLC-PDA chromatogram for (**A**) So(MW)-1, (**B**) So(Conv)-1, (**C**) So(Conv-2), (**D**) Ts(Conv)-1, and (**E**) Ts(Conv)-2 extracts (**1**—gallic acid; **2**—protocatechuic acid; **3**—caftaric acid; **4**—chlorogenic; **5**—caffeic acid; **6**—*trans–p*–coumaric acid; **7**—*trans*–ferulic acid; **8**—rutin hydrate; **9**—rosmarinic acid).

The reverse phase HPLC-PDA analysis was applied for the identification and quantification of up to nine compounds in polyphenolic extracts of common sage and wild thyme considering their retention times and the UV spectra similarity with that of standard compounds. The corresponding chromatograms of prepared polyphenolic extracts are presented in Figure 2. In all samples, rosmarinic acid was the most abundant compound with a concentration in the range of 25.712–49.975 mg/g extract (6.608–16.952 mg/g plant) for *Salvia officinalis* extracts and from 39.004 to 41.975 mg/g extract (4.940–5.570 mg/g plant) for *Thymus serpyllum* extracts. The lowest and highest amount of rosmarinic acid was obtained for So(MW)-1 and So(Conv)-2, respectively (Table 2), while in the case of Ts extracts, similar content for both ethanolic and hydroalcoholic extracts was identified. The amount of rosmarinic acid determined in So extracts is higher than that reported by Kozics (16.33 mg/g extract) for hydroalcoholic extracts [49] and similar or lower than that obtained by Farhat for methanolic extracts (13.68–18.34 mg/g plant) [50]. Additionally, a higher amount of rosmarinic acid was reported for *S. officinalis* ethanolic extract prepared at room temperature using ultrasounds for 15 min (45 ± 0.3 mg/g extract) [17] and for 75% aqueous methanolic extract of Ts (21.72 mg/g plant) [51].

Gallic acid was only identified in the So(Conv)-1 extract, rutin hydrate only in the So(MW)-1 extract and ferulic acid was found only in ethanolic extracts of sage prepared by both conventional extraction and MW treatment (Table 2). Although, Sonmezdag et al. found in 75% aqueous methanolic extract of Ts, gallic acid (0.63 mg/g plant) and ferulic acid (4.54 mg/g plant) besides rosmarinic, chlorogenic and protocatechuic acids, in our Ts extracts they cannot be quantified [51].

The content of protocatechuic acid in So extracts ranges between 0.069 and 0.200 mg/g extract (0.018–0.680 mg/g plant) and from 0.183 to 0.354 mg/g extract (0.022–0.051 mg/g plant) for Ts extracts with higher amounts in the case of using ethanol–water mixture as a solvent. One can notice that in the case So(MW)-1 extract, protocatechuic acid is detectable in comparison with the conventional extract. The amount of protocatechuic acid reported by Kozics et al. for common sage ethanolic–water extracts (v/v = 40/10) is lower than the one found in our extracts (0.010 mg/g extract) [49].

Caftaric acid was identified only in the *Salvia officinalis* extracts with an enhanced content when MW irradiation was applied or in the case of the use of ethanol–water mixture as solvent (0.360–0.821 mg/g extract or 6.8–28.1 mg/100 g plant). The content in our extracts was mostly higher than that reported by Oniga et al. for So 70% ethanolic extracts prepared at 60 °C (10.37 mg/100 g plant) [44].

Chlorogenic acid concentration, as in the case of protocatechuic acid, increased with the addition of water in the extraction mixture for either So or Ts extracts, this compound being found in all analyzed samples. The concentrations found in So extracts (0.286–0.870 mg/g extract or 0.074–0.297 mg/g plant) were higher than in the case of Ts extracts (0.320–0.775 mg/g extract or 0.038–0.111 mg/g plant).

The amount of caffeic acid found in So extracts ranged between 0.813 and 1.623 mg/g extract (0.209–0.555 mg/g plant) and for Ts between 1.043 and 1.553 mg/g extract (0.123–0.222 mg/g plant). The caffeic acid amount for So extracts are higher than that of Kozics (0.68 mg/g extract) for hydroalcoholic extracts [49], but similar or slightly lower than of Farhat for methanolic extracts (0.222–0.695 mg/g plant) [50].

*Trans-p*-coumaric and *trans*-ferulic acids were only detected in the So ethanolic extracts with amounts ranging from 0.015 and 0.019 mg/g plant and 0.134–0.180 mg/g extract (both having 0.034 mg/g plant), respectively, which are lower than the values obtained by Farhat for methanolic extract (0.312–0.703 mg/g plant) [50].

Rutin hydrate was detectable only in So(MW)-1 extract with a relatively high content (0.604 ± 0.000 mg/g extract or 0.155 ± 0.000 mg/g plant), which can explain the best antioxidant activity of the extract in comparison with all other extracts even if the extract contains the lowest amount of rosmarinic acid.

### 3.3. Radical Scavenger Activity of Polyphenolic Extracts

The radical scavenger activity (RSA) of sage extracts determined by both DPPH and ABTS assays were in the range of 180.81–236.43 mg TE/g extract (3.42–7.08 g TE/100 g plant) and 96.81–232.71 mg TE/g extract (1.83–5.98 g TE/100 g plant), respectively. The Ts extracts exhibited lower RSA values than So extracts, 161.61–185.89 mg TE/g extract and 1.90–2.65 g TE/100 g plant—DPPH method, and in the range of 74.44–105.63 mg TE/g extract or 0.88–1.51 g TE/100 g plant—ABTS method (Table 1). The values of radical scavenging activity of our So extracts were higher than that reported by Fernandes et al. for an acetone/water/acetic acid = 70/28/2 (v/v/v) extract obtained at 4 °C [52]. Additionally, Pasca et al. reported values of RSA determined by ABTS method of 123 ± 1 µmol TE/g extract and 126 ± 2 µmol TE/g extract for ethanolic extracts of So and Ts, respectively [42], which are lower than our values for So and Ts ethanolic extracts (188–330 µmol TE/g extract and 145 ± 2 µmol TE/g extract, respectively).

The RSA of polyphenolic extracts was assessed by DPPH assay. Three concentrations were chosen from the linearity domain to determine the concentration that can inhibit 50% of DPPH free radicals (IC50%). The values are listed in Table 3 and compared to that of caffeic and rosmarinic acids, two phenolic compounds presented in all prepared extracts. One can notice that, So extracts presented higher radical scavenger activity than Ts extracts, the best one being So(MW)-1. However, an enhanced antioxidant activity was obtained when ethanol–water mixture was employed as extraction solvent.

### 3.4. Antimicrobial Activity of Polyphenolic Extracts

The antimicrobial properties of polyphenolic extracts from common sage and wild thyme were assessed according to the standardized value of the positive control (15 mm for gentamycin and 17 mm for fluconazole) against nine reference bacterial and two fungus strains. Table 4 lists the values for inhibition growth zone diameter values in mm for So(MW), So(Conv)-2, and Ts(conv)-2 determined by disk diffusion method, as well as MIC and MBC through broth dilution assay against strains for which large diameters of microbial growth inhibition by the disk diffusion method were obtained. As it can be noticed, *Salvia officinalis* extracts exhibited better antimicrobial activity than wild thyme ones. The common sage extracts were active against all tested bacterial strains, the highest values of inhibition zone diameter and lowest values for MIC and MBC being obtained for *Staphylococcus aureus* ATCC 25923*, Streptococcus pneumoniae* ATCC 49619, and *Streptococcus pyogenes* ATCC 19615, while for wild thyme extract, the best results were obtained for the two types of *Streptococcus* strains. All polyphenolic extracts exhibited low antifungic activity against *Candida albicans* ATCC 10231 and *Candida parapsilosis* ATCC 22019. Unlike the common sage extracts, Ts(Conv)-2 exhibited significant lower antimicrobial activity against *Staphylococcus aureus* ATCC 25923.

### 3.5. Characterisation of Mesoporous Supports

As supports for embedding the polyphenolic extracts, two inorganic mesoporous materials, MCM-41 silica and titania, were employed. The supports obtained by the sol–gel method were characterized by small- and wide-angle XRD to assess their structural features, FTIR spectroscopy and TG analysis to evidence the removal of the structure directing agents used in their synthesis, N_2_ sorption measurements to evaluate their porosity, as well as SEM and TEM to investigate the morphology.

**Figure 3:**
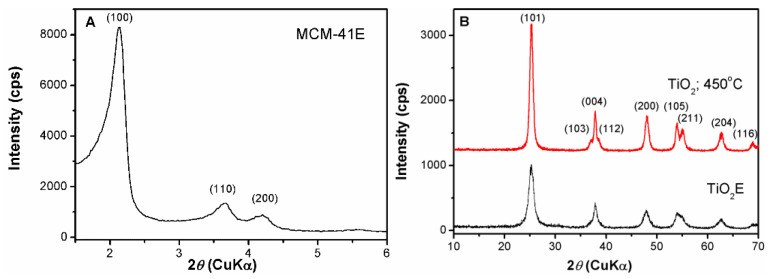
(**A**) Small-angle XRD analysis of MCM-41E and (**B**) wide-angle XRD patterns of TiO_2_E and TiO_2_ calcined at 450 °C.

The small-angle XRD pattern of MCM-41E silica support demonstrated the formation of an ordered hexagonal pore array, which belongs to the *P6m* symmetry. The sample exhibited three Bragg reflections (100), (110), and (200) characteristic for mesostructured MCM-41-type materials (Figure 3A). Unlike silica, titania support has no ordered pore framework, but it is crystalline having anatase structure with tetragonal symmetry (ICDD 21-1272) as wide-angle X-ray diffraction analysis proved (Figure 3B). The sol–gel process of titanium alkoxides used as precursors, differs from the one of TEOS because of higher chemical reactivity resulted from the lower electronegativity of titanium than silicon and its ability to enhance spontaneously its coordination number with water molecules. The hydrolysis rate of titanium isopropoxide, much faster than of TEOS, hinders the cooperative assembly of inorganic species with the surfactant molecules and thus, for titania material is difficult to obtain an ordered pore array.

The anatase structure was formed after the ageing treatment of the reaction mixture at reflux for 24 h that was evidenced by wide-angle XRD pattern. The template agent, Pluronic P123, interacted with titania nanoparticles surface as it was not completely removed after Soxhlet extraction in ethanol for 30 h as both FTIR spectroscopy and TG analysis demonstrated. In the FTIR spectrum of titania sample recovered after Soxhlet extraction, TiO_2_E, one can notice the characteristic bands of the anatase matrix, ν(Ti–O) and ν(Ti–O–Ti) from 670 cm^−1^ and 478 cm^−1^, respectively, the stretching mode, ν(OH) of Ti–OH groups (3000–3600 cm^−1^) and the δ(HOH) band (1640 cm^−1^) [53], besides the weak vibrations of the structure directing agent in 2800–2950 cm^−1^ and 1410–1530 cm^−1^ domains, demonstrating the presence of copolymer traces on the surface of titania nanoparticles (Supplementary Material, Figure S1). The TG analysis of TiO_2_E showed a total weight loss of 8.8% in 25–450 °C temperature range, higher than for TiO_2_ calcined at 450 °C, which presented a weight loss of 2.2 wt% up to 150 °C that can be ascribed to the removal of water molecules physically adsorbed (Supplementary Material, Figure S2). The calcining step at 450 °C, 5 h led to the anatase phase preservation, an increase of its crystallinity (the diffraction peaks being well defined) and of the crystallite size from 7 for TiO_2_E sample to 11 nm for the calcined material (Figure 3B), determined using Rigaku PDXL software 1.8 (Rigaku Corporation, Tokyo, Japan, 2006) based on Scherrer’s equation considering (101) Bragg reflection.

**Figure 4:**
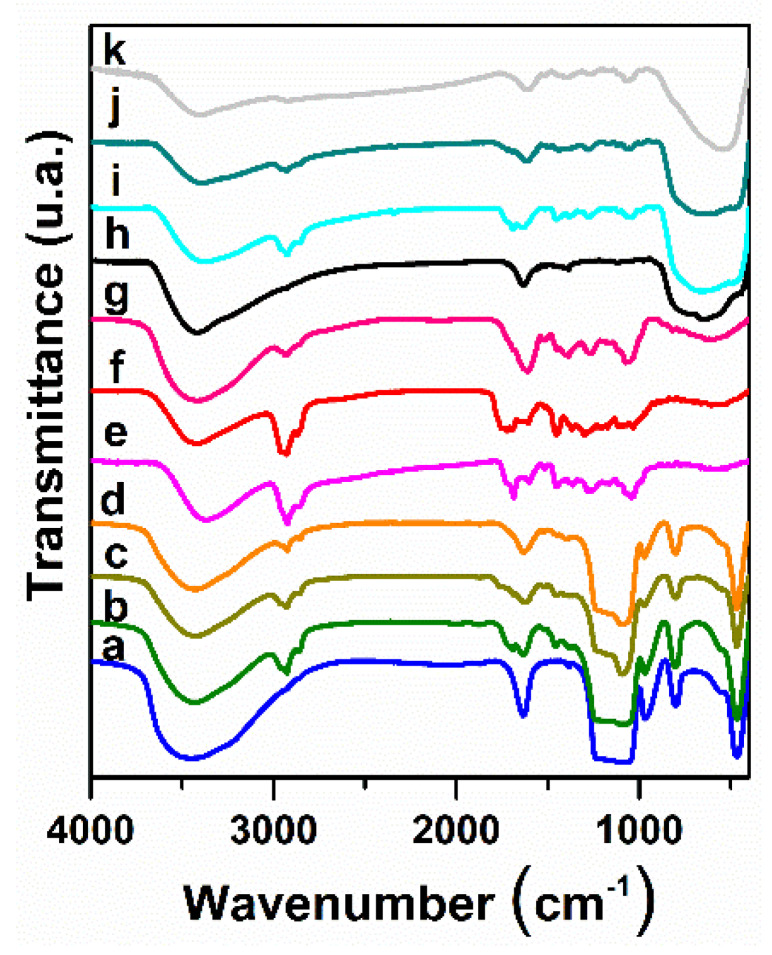
FTIR spectra for (a) MCM-41E; (b) So(MW)-1@MCM-41E; (c) So(Conv)-2@MCM-41E; (d) Ts(Conv)-2@MCM-41E; (e) So(MW)-1; (f) So(Conv)-2; (g) Ts(Conv)-2; (h) TiO_2_; (i) So(MW)-2@TiO_2_; (j) So(Conv)-2@TiO_2_; and (k)Ts(Conv)-2@TiO_2_.

The FTIR spectrum of the MCM-41E material evidenced the characteristic bands of silica matrix: asymmetric and symmetric stretching vibrations of Si–O–Si bonds at 1096 cm^−1^ and 814 cm^−1^, respectively, the band of silanol groups at 964 cm^−1^ and the bending vibrations of Si–O–Si bonds at 471 cm^−1^, as well as the broad envelope in the range 3000–3600 cm^−1^ assigned to the associated hydroxyl groups through hydrogen bonds and the bending vibration at 1630 cm^−1^ of water adsorbed into the mesopores (Figure 4, curve a) [34]. The absence of the structure directing agent demonstrated its complete removal by extraction process. In the case of titania support, the template agent was removed by calcination at 450 °C for 5 h, which was proved by FTIR spectrum (Figure 4, curve h) that showed only the characteristic vibrations of anatase matrix.

**Figure 5:**
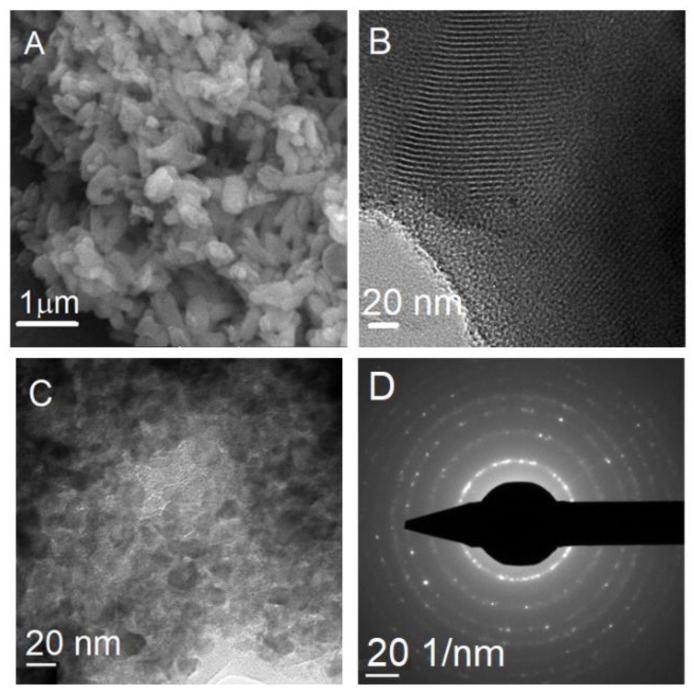
SEM image of (**A**) MCM-41E material; (**B**) TEM micrograph of MCM-41E material; (**C**) TEM image of TiO_2_; and (**D**) SAED analysis of TiO_2_ support.

SEM analysis of MCM-41E material revealed the formation of either spherical particles with a diameter in the range of 200−400 nm or short rods of 1−1.5 μm length (Figure 5A), while TEM investigation demonstrated the presence of an ordered pore framework with long channels of mesopores (Figure 5B) in accordance with small-angle XRD data. TEM investigation of TiO_2_ support showed the formation of polyhedral nanoparticles with uniform size, in the range of 11−15 nm in agreement with the crystallite size determined from XRD analysis and small pores between nanoparticles (Figure 5C), while selected area electron diffraction (SAED) proved the crystalline nature of the material (Figure 5D).

**Figure 6:**
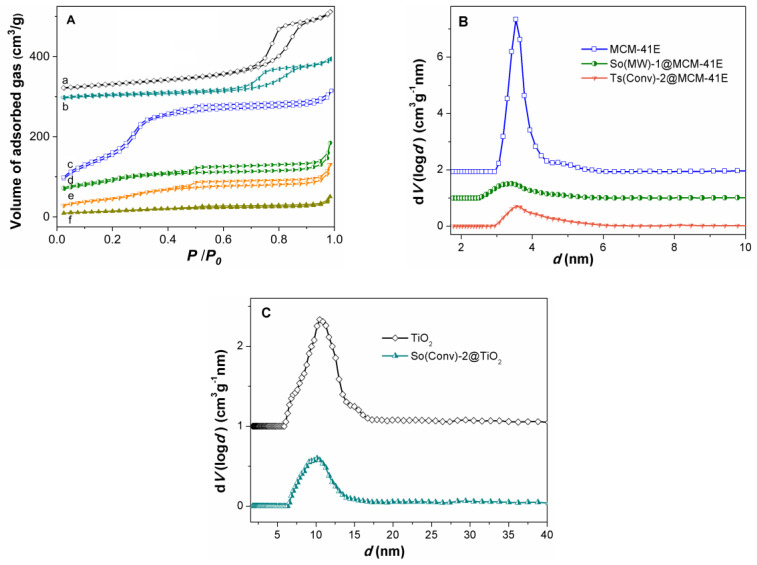
(**A**) N_2_ adsorption–desorption isotherms for: (a) TiO_2_; (b) So(Conv)-2@TiO_2_; (c) MCM-41; (d) So(MW)-1@MCM-41E, (e) Ts(Conv)-2@MCM-41E, and (f) So(Conv)-2@MCM-41E. (**B**) Pore size distribution curves for MCM-41E support, So(MW)-1@MCM-41E, and Ts(Conv)-2@MCM-41E. (**C**) Pore size distribution curves for TiO_2_ and So(Conv)-2@TiO_2_.

In order to be used as matrices for embedding the polyphenolic extracts, the synthesized materials must have porosity to host a large amount of natural compounds. The nitrogen adsorption–desorption isotherms of TiO_2_ and MCM-41E supports show high porosity being type IV according IUPAC classification, which are characteristic for mesoporous materials (Figure 6A, curve a,c). The textural features, specific surface area, *S*_BET_, total pore volume, *V*_p_, and the average pore diameter computed with non-local density functional theory (NLDFT) from the adsorption branch of isotherm, *d*, for supports were listed in Table 5. As expected MCM-41E material had larger values of *S*_BET_ and *V*_p_ (689 m^2^/g and 0.54 cm^3^/g, respectively) than TiO_2_ (115 m^2^/g and 0.34 cm^3^/g, respectively), which exhibited interparticles porosity. The N_2_ adsorption–desorption isotherm of MCM-41E material was almost reversible with a sharp increase in adsorbed gas volume in 0.2−0.35 relative pressure range (Figure 6A, curve d) that corresponds with a narrow pore size distribution curve. For titania nanomaterial, an increase in the volume of adsorbed gas at 0.7 relative pressure on the isotherm adsorption branch, and a fast desorption associated with capillary nitrogen condensation could be observed (Figure 6A), which was associated with a larger pore diameter (10.49 nm) than that of the MCM-41E material (3.54 nm).

### 3.6. Characterization of Materials Containing Extract

To enhance the polyphenolic extracts stability and thus, to preserve their radical scavenger and antimicrobial properties, they were embedded into mesoporous inorganic materials. As supports for polyphenolic extracts loading, MCM-41E silica and TiO_2_, were chosen due to their capacity to accommodate organic molecules. For encapsulation of polyphenolic extracts from common sage or wild thyme, the incipient wetness impregnation procedure was applied, which consists of vacuum drying of support, mixing the support with the polyphenolic extract, and drying in vacuum of obtained suspension. To compare the effect of support nature, a similar amount of a polyphenolic extract was loaded either in MCM-41E or TiO_2_, although silica support had higher porosity and it was able to accommodate higher amount of phytochemicals than titania. By overcoming the surface interactions that govern the adsorption method, which make the quantity of the compounds adsorbed very sensitive at various parameters, the incipient wetness impregnation procedure allows a very good control of the amount of phytochemicals adsorbed physically into the matrix mesopores that is limited by its total pore volume [29].

The obtained extract-loaded materials were characterized by FT-IR spectroscopy to evidence the presence of phytochemicals, nitrogen adsorption–desorption isotherms to demonstrate that natural compounds filled the mesopores of inorganic support, and thermal analysis to determine the content of natural compounds in materials containing extract. Additionally, the radical scavenger activity and antimicrobial properties were assessed.

In the FTIR spectra of extract-loaded materials (Figure 4, curve b–d,i–k), it can be noticed vibrations specific to both extract and corresponding support. The stretching vibrations of C−H bonds in the 2800−3000 cm^−1^ region, the stretching vibrations of C–O bond (1695–1714 cm^−1^) and skeletal =C–O–C vibrations (1500–1524 cm^−1^) were ascribed to polyphenolic compounds.

The nitrogen adsorption–desorption isotherms confirmed the filling of support mesopores with polyphenols (Figure 6A, curve b,d–f). However, a low porosity remained after the extract loading, the total pore volume ranging from 0.17 to 0.24 cm^3^/g. The average pore size decreased in comparison with the corresponding support, which confirmed the pores loading with polyphenolic compounds (Table 5 and Figure 6B,C).

The content of polyphenolic compounds in materials containing extract was determined by thermal analysis (TG-DSC) considering the total weight loss up to 500 °C (materials based on TiO_2_) or 600 °C (samples containing MCM-41E support), after subtraction the weight loss of physically adsorbed water molecules, which corresponds to the first endothermic event (Supplementary Material, Figure S3), and also considering the extract residue due to chlorophyll content (Supplementary Material, Figure S4 and Figure S5). The polyphenolic content was in the range of 22–29 wt % for So(MW)-1 extract and 20–22 wt %, for Ts(Conv)-2. Due to higher porosity of MCM-41E support than of TiO_2_, a larger amount of phenolic compounds from So(Conv)-2 extract was loaded on MCM-41E support (44 wt %) than into titania mesopores (22 wt %) to study if the properties of natural compounds were preserved (Table 4).

### 3.7. Recovery of Components from Materials Containing Extract

To demonstrate the possibility of recovering the components of materials containing embedded extract, So(Conv)-2@MCM-41E sample was mixed with ethanol–water (4/1 v/v) for 24 h, having a content of 20 mg/mL, in a closed container, at room temperature, under dark conditions, followed by centrifugation to separate the components. The recovered extract was analyzed by HPLC-PDA (Supplementary Material, Figure S6), and the same five substances were identified and quantified (protocatechuic acid: 0.224 ± 0.005 mg/g extract; caftaric acid: 0.801 ± 0.031 mg/g extract; chlorogenic acid: 0.878 ± 0.009 mg/g extract; caffeic acid: 1.474 ± 0.065 mg/g extract; and rosmarinic acid: 45.756 ± 0.537 mg/g extract), showing a 92% recovery yield. Additionally, the inorganic support was recuperated with a high yield (90%). The properties of MCM-41E material were not altered during the extract adsorption–desorption processes, its ordered pore array being preserved as small-angle XRD pattern proved (Figure S7). The almost total recovery of phenolic substances from silica support was also demonstrated by FTIR spectrum of MCM-41E recuperated from material containing extract, in which the characteristic bands of phenolic compounds were hardly seen (Figure S8).

### 3.8. Radical Scavenging Capacity of Materials Containing Extract

**Figure 7:**
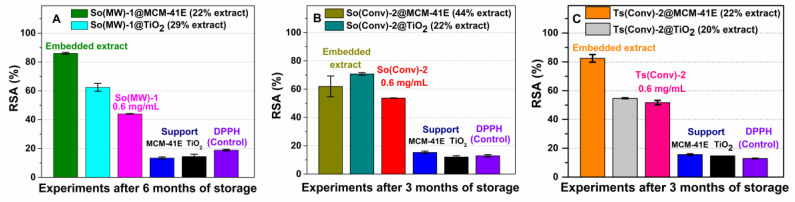
In vitro radical scavenger activity for (**A**) So(MW)-1 extract embedded in MCM-41E or TiO_2_; (**B**) So(Conv)-2 extract embedded both supports; and (**C**) material containing Ts(Conv)-2, in comparison with the corresponding extract and support.

The efficiency of materials containing extract was further tested as they would be employed in future applications. Therefore, the radical scavenger activity of materials containing extract was assessed using a method that we previously reported (DPPH assay), which compares in the same time the antioxidant activity of material containing embedded extract with that of the free extract and support at the same concentration as in the embedded extract, after 24 h, using as a control the degradation of the DPPH free radical solution [29]. One can consider that the preservation of radical scavenger activity in time means that the extract is stable. A higher stability was achieved (considering RSA) by loading polyphenols into mesoporous supports than that of the free extracts after 3–6 months of a storage at 4 °C. At similar polyphenols content into mesoporous support, the loading of common sage or wild thyme extracts into MCM-41E mesopores led to a higher RSA in comparison with TiO_2_ (Figure 7A,C), while in the case of So(Conv)-2@MCM-41E (with higher content of polyphenolic compounds, 44 wt %) the RSA was lower than of So(Conv)-2@TiO_2_ (Figure 7B). This could be explained that in the case of MCM-41E, the support pores were completely occupied by extract compounds and a loss of mesoporous matrix protection against external factors was achieved. The supports did not exhibit a noticeable antioxidant activity and from the RSA of materials containing extract, one can confirm that a higher stability was accomplished for extract-loaded materials that still exhibited some porosity after the extract embedding. 

All materials containing extracts showed improved stability in comparison with the free extracts and hence conferred the possibility of developing cosmetical or nutraceutical formulations.

### 3.9. Antimicrobial Properties of Materials Containing Extract

The antimicrobial properties of selected extract-loaded material, So(Conv)-2@MCM-41E, was tested on the same nine bacterial and two fungus strains in the same conditions as the free extract by the disk diffusion method on solid sample. The values for inhibition growth zone diameter measured in mm are listed in Table 6. Slightly larger diameters of inhibition growth zone than for the corresponding So(Conv)-2 free extract were obtained for all tested strains. The same type of elicited activity for the extract could be observed also for the extract-loaded material, namely the best bactericidal activity against *Staphylococcus aureus* ATCC 25923*, Streptococcus pneumoniae* ATCC 49619, and *Streptococcus pyogenes* ATCC 19615 strains.

## 4. Conclusions

The effect of polyphenolic compounds nanoconfinement into mesopores of silica and titania nanomaterials on their antioxidant capacity and antimicrobial potential was studied.

Polyphenolic ethanolic and ethanol–water (4/1 v/v) extracts from *Salvia officinalis* L. and *Thymus serpyllum* L. were prepared by either conventional extraction or the MW-assisted process. All extracts exhibited radical scavenging capacity determined by both the DPPH assay (180.81–236.43 mg TE/g extract for common sage extracts and 161.61–185.59 mg TE/g extract for wild thyme extracts) and ABTS method (96.81–232.71 mg TE/g extract for So extracts and 74.44–105.63 mg TE/g extract for Ts extracts). A higher radical scavenger activity was observed when the ethanol–water mixture was used as a solvent or in the case of MW-assisted extraction, which can be correlated with the total polyphenols content of extracts. Additionally, all the screened extracts demonstrated good bactericidal activity against tested reference strains, especially in the case of *Staphylococcus aureus* ATCC 25923*, Streptococcus pneumoniae* ATCC 49619, and *Streptococcus pyogenes* ATCC 19615. However, common sage extracts exhibited either higher antioxidant capacity or better antimicrobial potential than wild thyme extracts.

The chemical profile of polyphenolic extracts was determined by HPLC-PDA analysis. The main compound found in common sage and wild thyme extracts was rosmarinic acid. The other four polyphenolic substances, protocatechuic acid, caftaric acid, chlorogenic acid, and caffeic acid, were identified and quantified in all prepared extracts. Rutin hydrate was identified only in So(MW)-1 extract; this could explain its higher radical scavenger activity compared to the other tested extracts.

In order to improve the stability of polyphenolic extracts and hence, to preserve their radical scavenging and antimicrobial properties in time, the extracts were loaded into mesoporous inorganic nanomaterials. As matrices for loading polyphenolic extracts, mesoporous MCM-41 silica and titania nanoparticles were chosen. The inorganic supports prepared by the sol–gel method exhibited porosity being able to host polyphenolic compounds in their mesopores. The embedded extracts presented higher radical scavenger activity than that of the free extracts after 3–6 months storage at 4 °C, which could indicate a better stability of extract when loaded into mesoporous materials. Regarding the RSA of materials containing natural compounds, better results were observed for MCM-41E support, when matrix mesopores were not filled completely with phytochemicals. Additionally, the tested material containing extract exhibited very good antimicrobial activity against nine reference strains, the inhibition zone diameters being slightly larger for all tested bacteria.

The advantages of using mesoporous materials for loading polyphenolic extracts from *Salvia officinalis* L. and *Thymus serpyllum* L was demonstrated. All materials containing polyphenolic extract showed enhanced stability and antimicrobial properties than that of the free extracts and thus, this new approach could be further employed to develop cosmetics or nutraceutical formulations based on nanomaterials.

## Figures and Tables

**Table 1:** Total polyphenols content, radical scavenger activity, total flavonoids, and chlorophyll a and b pigments content for the polyphenolic extracts.

Extract	Extract (wt %)	TPC (mg_CAE_/g_e_)	TPC (mg_GAE_/g_e_)	RSA_DPPH_ (mg_TE_/g_e_)	RSA_ABTS_ (mg_TE_/g_e_)	TFC (mg_RE_/g_e_)	Ch-a (mg_Ch-a_/g_e_)	Ch-b (mg_Ch-b_/g_e_)
So(MW)-1	25.70	107.45 ± 3.53	119.85 ± 3.94	236.43 ± 1.77	232.79 ± 8.23	75.52 ± 0.70	3.95 ± 0.46	0.97 ± 0.11
So(Conv)-1	18.89	97.68 ± 1.53	108.95 ± 1.70	180.81 ± 5.75	96.81 ± 3.63	70.17 ± 0.37	3.12 ± 0.03	0.57 ± 0.02
So(Conv)-2	34.19	164.54 ± 3.71	181.11 ± 4.07	215.74 ± 13.72	169.99 ± 5.52	40.92 ± 1.02	0.64 ± 0.02	0.29 ± 0.03
Ts(Conv)-1	11.77	140.51 ± 4.32	154.67 ± 4.75	161.61 ± 15.58	74.44 ± 0.92	83.10 ± 1.85	5.25 ± 0.00	0.91 ± 0.03
Ts(Conv)-2	14.28	141.24 ± 3.26	155.50 ± 3.61	185.89 ± 2.76	105.63 ± 4.56	81.28 ± 2.03	1.05 ± 0.04	0.28 ± 0.02

TPC—total polyphenols content as caffeic acid equivalents (CAE) and gallic acid equivalents (GAE); RSA—radical scavenger activity, TE—Trolox equivalents; TFC—total flavonoids content (RE—rutin hydrate equivalents); Ch-a and Ch-b—chlorophyll a and b pigments content. All data are expressed in mg/g extract.

**Table 2:** Identification and quantification of polyphenolic compounds by reverse phase HPLC-PDA.

	Concentration in Extract (mg/g extract)					
Standard Compound	RT (min)	So(MW)-1	So(Conv)-1	So(Conv)-2	Ts(Conv)-1	Ts(Conv)-2
gallic acid	3.605	nd	0.028 ± 0.000	nd	nd	nd
protocatechuic acid	6.982	0.069 ± 0.000	nd	0.200 ± 0.001	0.183 ± 0.001	0.354 ± 0.023
caftaric acid	11.376	0.426 ± 0.001	0.360 ± 0.001	0.821 ± 0.002	nd	nd
chlorogenic acid	13.016	0.286 ± 0.000	0.340 ± 0.002	0.870 ± 0.000	0.320 ± 0.001	0.775 ± 0.009
caffeic acid	15.075	0.813 ± 0.001	1.505 ± 0.010	1.623 ± 0.006	1.043 ± 0.008	1.553 ± 0.022
*trans p*-coumaric acid	21.674	0.060 ± 0.001	0.102 ± 0.000	nd	nd	nd
*trans* ferulic acid	25.027	0.134 ± 0.000	0.180 ± 0.002	nd	nd	nd
rutin hydrate	25.964	0.604 ± 0.000	nd	nd	nd	nd
rosmarinic acid	32.139	25.712 ± 0.014	41.600 ± 0.253	49.582 ± 0.034	41.975 ± 0.028	39.004 ± 0.125

RT—retention time, nd—not detected. All concentrations are given in mg compound per gram of extract.

**Table 3:** Radical scavenging activity of polyphenolic extracts assessed by DPPH assay.

Sample	IC50% (mg/mL)	Correlation equation	R^2^
So(MW)-1	1.15	*y* = 43.321*x* + 0.326	0.9940
So(Conv)-1	1.50	*y* = 31.526*x* + 2.731	0.9974
So(Conv)-2	1.26	*y* = 34.660*x* + 6.447	0.9970
Ts(Conv)-1	1.68	*y* = 25.890*x* + 6.569	0.9980
Ts(Conv)-2	1.46	*y* = 30.116*x* + 6.079	0.9910
Caffeic acid	0.20	*y* = 239.815*x* + 3.130	0.9999
Rosmarinic acid	0.13	*y* = 383.122*x* + 1.780	0.9996

**Table 4:** Antimicrobial activity of polyphenolic extracts.

Strain Species	*Φ*_So(MW)_ (30 mg/mL)	MIC/MBC (mg/mL)	*Φ*_So(conv)-2_ (20 mg/mL)	MIC/MBC (mg/mL)	*Φ*_Ts(conv)-2_ (28.5 mg/mL)	MIC/MBC (mg/mL)
*Salmonella enterica* serotip typhimurium	17	7.5/15	10	-	9	-
*Shigella flexneri* serotype 2b	16	7.5/15	10	-	9	-
*Enterococcus faecalis*	18	7.5/7.5	18	10.125/10.125	10	-
*Escherichia coli*	17	7.5/7.5	18	10.125/10.125	10	-
*Pseudomonas aeruginosa*	17	7.5/15	11	-	10	-
*Staphylococcus aureus*	19	3.75/7.5	19	5.06/10.125	11	-
*Streptococcus pneumoniae*	20	3.75/7.5	19	5.06/10.125	15	14.25/28.5
*Streptococcus pyogenes*	22	3.75/7.5	20	5.06/10.125	17	14.25/28.5
*Bacteroides fragillis*	17	7.5/7.5	18	10.125/10.125	9	-

*Φ*—inhibition growth zone diameter in mm for polyphenolic extracts in mentioned concentration; MIC—minimum inhibitory concentration; and MBC—minimum bactericidal concentration.

**Table 5:** Textural features for supports and materials containing embedded extracts.

Support	*d* (nm)	*S_BET_* (m^2^/g)	*V_p_* (cm^3^/g)	Embedded Extract	Extract (wt %)	*d* (nm)	*V_p_* (cm^3^/g)
TiO_2_	10.49	115	0.34	So(MW)-1@TiO_2_	29	-	-
				So(Conv)-2@TiO_2_	22	10.13	0.16
				Ts(Conv)-2@TiO_2_	20	-	-
MCM-41E	3.54	689	0.54	So(MW)-1@MCM-41E	22	3.42	0.20
				So(Conv)-2@MCM-41E	44	-	0.08
				Ts(Conv)-2@MCM-41E	22	3.54	0.18

**Table 6:** Antimicrobial activity of So(Conv)-2@MCM-41E.

Species Strains	*Φ* (mm)
*Salmonella enterica*	11
*Shigella flexneri* serotype 2b	11
*Enterococcus faecalis*	23
*Escherichia coli*	21
*Pseudomonas aeruginosa*	14
*Staphylococcus aureus*	23
*Streptococcus pneumoniae*	23
*Streptococcus pyogenes*	23
*Bacteroides fragillis*	21
*Candida albicans*	9
*Candida parapsilosis*	9

*Φ*—inhibition growth zone diameter.

## Supplementary Materials

The following are available online at https://www.mdpi.com/2079-4991/10/5/820/s1, Figure S1. FTIR spectrum of TiO_2_E; Figure S2. TG analysis of titania samples; Figure S3. Thermal analysis for A-So(MW)-1@MCM-41E, B-So(Conv)-2@TiO_2_, C-Ts(Conv)-2@MCM-41E, and D-Ts(Conv)-2@TiO_2_; Figure S4. Thermal analysis (TG-DSC) of dried So(MW)-1 extract; Figure S5. SEM micrograph of solid residue of So(MW)-1 extract burnt at 900 °C; Figure S6. HPLC-PDA analysis of recovered common sage extract in ethanol–water mixture from So(Conv)-2@MCM-41E material after 24 h; Figure S7. Small-angle XRD pattern of recovered MCM-41E support in ethanol–water mixture from So(Conv)-2@MCM-41E material after 24 h; Figure S8. FTIR spectrum of recovered MCM-41E support in ethanol–water mixture from So(Conv)-2@MCM-41E material after 24 h.
