# Supplementary material for: Properties of Salvia officinalis L. and Thymus serpyllum L. Extracts Free and Embedded into Mesopores of Silica and Titania Nanomaterials
Source: Nanomaterials (Basel). 2020 Apr 25;10(5):820. doi: 10.3390/nano10050820 (PMC7712395; doi:10.3390/nano10050820)
Supplement: Supplementary file 1 [file nanomaterials-10-00820-s001.pdf]

Supplementary Information

Properties of *Salvia Officinalis* L. and *Thymus Serpyllum* L. Extracts Free and Embedded into Mesopores of Silica and Titania Nanomaterials

Ana-Maria Brezoiu [1,†], Mioara Prundeanu [1,†], Daniela Berger [1,†], Mihaela Deaconu [1], Cristian Matei [1], Ovidiu Oprea [1], Eugeniu Vasile [2,*], Ticuța Negreanu-Pîrjol [3], Delia Muntean [4], Corina Danciu [5]

[1] Department of Inorganic Chemistry, Physical-Chemistry & Electrochemistry, Faculty of Applied Chemistry and Materials Science, University "Politehnica" of Bucharest, 1-7 Gheorghe Polizu Street, 011061, Bucharest, Romania; anamaria_brezoiu@yahoo.com (A.-M.B.); mioara_prundeanu@yahoo.com (M.P.); daniela.berger@upb.ro (D.B.); mihaela_deaconu@yahoo.com (M.D.); cristian.matei@upb.ro (C.M.); ovidiu.oprea@upb.ro (O.O.)
[2] Department of Oxide Materials Science and Engineering, Faculty of Applied Chemistry and Materials Science, University "Politehnica" of Bucharest, 1-7 Gheorghe Polizu Street, 011061, Bucharest, Romania
[3] Faculty of Pharmacy, "Ovidius" University of Constanta, Aleea Universitatii No. 1, Constanta 900470, Romania; ticuta_np@yahoo.com
[4] Department of Microbiology, University of Medicine and Pharmacy "Victor Babes", Eftimie Murgu Square No. 2, 300041 Timisoara, Romania; muntean.delia@umft.ro
[5] Department of Pharmacognosy, University of Medicine and Pharmacy "Victor Babes", Eftimie Murgu Square No. 2, 300041 Timisoara, Romania; corina.danciu@umft.ro
* Correspondence: eugeniuvasile@yahoo.com_
† These authors contribute equally to this work.

Received: 31 March 2020; Accepted: 21 April 2020; Published: 25 April 2020

1. Characterization of mesoporous supports

The FTIR spectrum of titania sample purified by Soxhlet extraction in ethanol, TiO_2E, shows the presence of traces of structure directing agent, triblock copolymer Pluronic P123, its characteristic vibrations being observed in 1410-1530 cm^{-1} domain (Figure S1). By comparing the TG curve of TiO_2E with that of TiO_2 material calcined at 450 °C, one can notice a higher weight loss (8.8% wt) in the case of TiO_2E than of calcined sample (2.2%wt) that can be attributed to the presence of template agent traces on the titania nanoparticles surface (Figure S2).

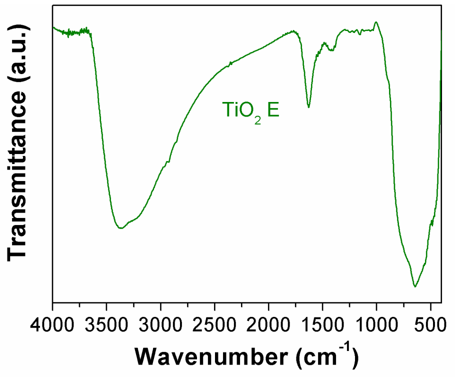

Figure S1. FTIR spectrum of TiO_2E.

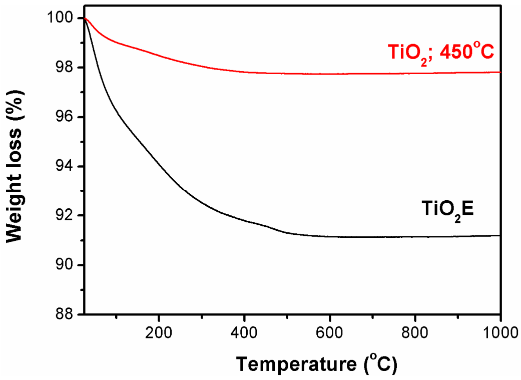

Figure S2. TG analysis of TiO_2 samples.

2. Thermal analysis (TG-DSC) of materials containing extracts

The amount of polyphenolic substances in the materials containing embedded extracts was determined by thermal analysis (TG-DSC or TG-DTA) neglecting the weight loss associated with the first endothermic event of DSC/DTA curve that can be attributed to the solvent molecules desorption (Figure S3), and also, considering the residue of extract due to chlorophyll content (Figure S4).

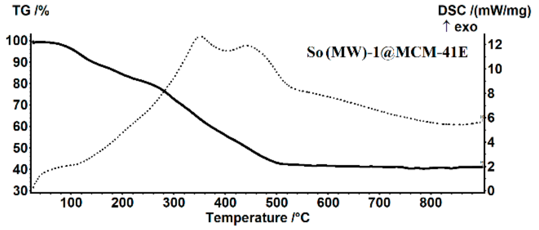

A

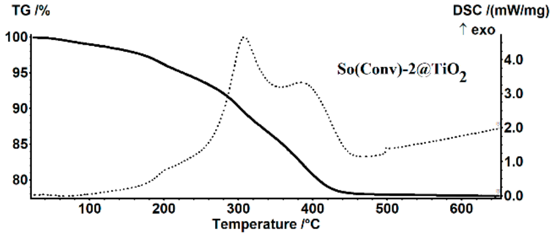

B

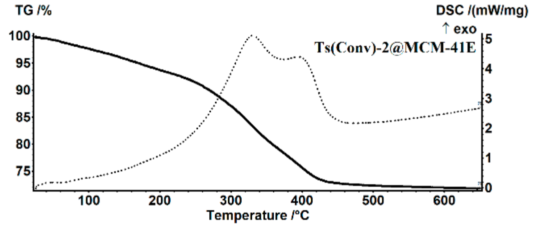

C

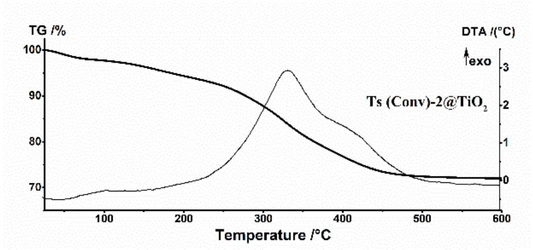

D

Figure S3. Thermal analysis for (**A**) So(MW)-1@MCM-41E, (**B**) So(Conv)-2@TiO_2, (**C**) Ts(Conv)-2@MCM-41E, and (**D**) Ts(Conv)-2@TiO_2.

After performing the thermal analysis of dried sage extract (Figure S4), the solid residue of So(MW)-1 (1.54% wt) was investigated by EDX analysis coupled to SEM. It was proved that the residue contained the following elements Mg, Si, P, S Ca, K and Mn (Figure S5), as expected due to the chlorophyll content, the main component being magnesium (18.5% wt).

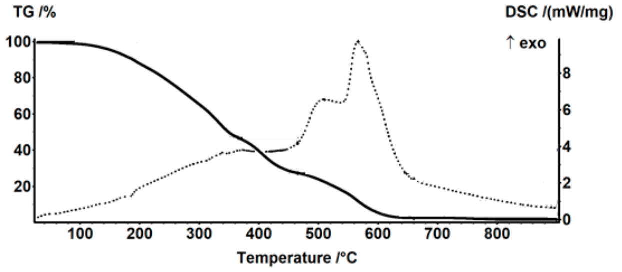

Figure S4. Thermal analysis (TG-DSC) of dried So(MW)-1 extract.

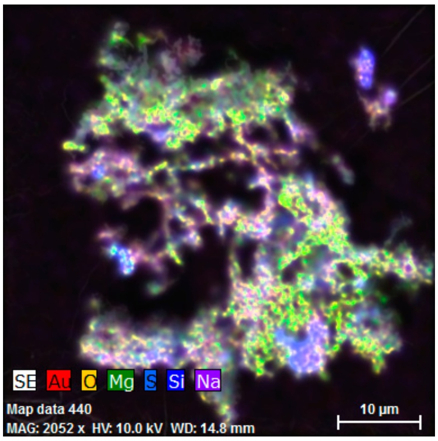

Figure S5. SEM-EDX elemental mapping of solid residue of So(MW)-1 extract burnt at 900 °C.

3. Recovery of components from materials containing embedded extract

The sage extract, So(Conv)-2, was recovered from So(Conv)-2@MCM-41E material by mixing the embedded extract with ethanol-water mixture (ethanol/water = 4/1 v/v) at room temperature, under constant magnetic stirring. After 24 h, the solid was removed by centrifugation, and the liquid was analysed by HPLC-PDA (Figure S6). In the sage extract recovered from So(Conv)-2@MCM-41E sample, the following polyphenols were identified and quantified: 1- protocatechuic acid: 0.224 ± 0.005 mg/g extract; 2 – caftaric acid: 0.801 ± 0.031 mg/g extract; 3 – chlorogenic acid: 0.878 ± 0.009 mg/g extract; 4 – caffeic acid: 1.474 ± 0.065 mg/g extract; 5 – rosmarinic acid: 45.756 ± 0.537 mg/g extract.

Also, the MCM-41E support was analysed by small-angle XRD and FTIR analyses. The small-angle XRD pattern of recovered MCM-41E support proved that the ordered pore array is not altered during the extract adsorption-desorption processes (Figure S7). The almost total recovery of phenolic compounds from pores of inorganic matrix was also proved by FTIR analysis of MCM-41E support after its recuperation from material containing embedded extract (Figure S8).

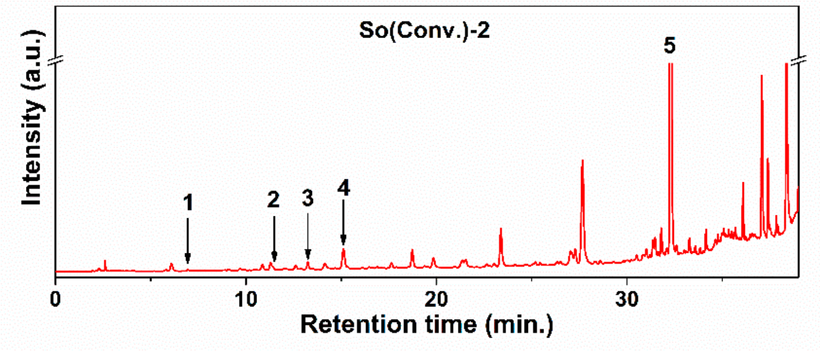

Figure S6. HPLC-PDA analysis of recovered common sage extract in ethanol-water mixture from So(Conv)-2@MCM-41E material after 24 h. (1 – protocatechuic acid; 2 – caftaric acid; 3 – chlorogenic acid; 4 – caffeic acid; 5 – rosmarinic acid).

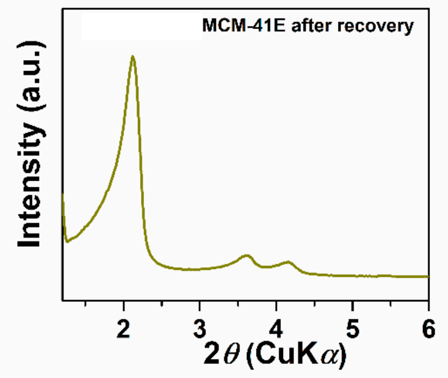

Figure S7. Small-angle XRD pattern of recovered MCM-41E support in ethanol-water mixture from So(Conv)-2@MCM-41E material after 24 h.

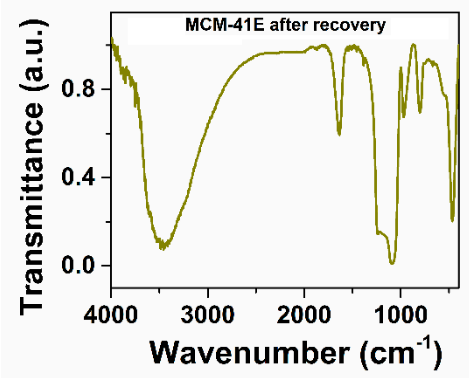

Figure S8. FTIR spectrum of recovered MCM-41E support in ethanol-water mixture from So(Conv)-2@MCM-41E material after 24 h.

© 2020 by the authors. Licensee MDPI, Basel, Switzerland. This article is an open access article distributed under the terms and conditions of the Creative Commons Attribution (CC BY) license (http://creativecommons.org/licenses/by/4.0/).
